# Well-Being Measurement in Africa: A Systematic Review

**DOI:** 10.3390/bs16101772

**Published:** 2026-09-28

**Authors:** Angelina Wilson Fadiji, Nnana Samuel Uchendu, Marie P. Wissing

**Affiliations:** 1 Department of Psychology, Faculty of Health and Life Sciences, De Montfort University, Leicester LE1 9BH, UK; 2 Department of Educatiional Psychology, Faculty of Education, University of Pretoria, Pretoria 0002, South Africa; 3 Africa Unit of Transdisciplinary Health Research, Faculty of Health Sciences, North-West University, Potchefstroom-Campus, Potchefstroom 2531, South Africa; marie.wissing@nwu.ac.za

**Keywords:** systematic review, validation, psychometric properties, positive psychology, well-being instruments, Africa

## Abstract

The general narrative in well-being measurement is a lack of representation of instruments developed and validated in the African context. However, there is no existing review solely focused on Africa that clearly demonstrates the extent of progress in the field and the current methodological challenges. The present study aims to systematically review and collate evidence on the validation of well-being measures in Africa. Following the Preferred Reporting Items for Systematic reviews and Meta-Analyses (PRISMA) method, a total of 64 research articles were reviewed. The study’s findings showed steady growth in well-being measurement in the African context. However, this growth has not been without challenges that are stifling our general understanding and consensus on how well-being should be measured in Africa. A persistent research gap is the development of new, contextually relevant measures of well-being. Among the recurring methodological challenges were translation issues, poor model fit indices and the use of small samples. We recommend further testing of locally developed well-being measures and their incorporation into international surveys, as cross-country assessments can only yield meaningful insights when the items used adequately capture local experiences. Translation of existing measures is key in accessing hard-to-reach populations, with such data informing clinical and community-based interventions.

## 1. Introduction

The present study aimed to explore and compile evidence specific to well-being instruments developed or validated for use in Africa as part of the well-being studies in Africa project, which is concerned with integrating evidence on the progress of well-being on the continent (Fadiji et al., 2024; Wilson Fadiji & Khumalo, 2026). These reviews highlight the limited understanding or knowledge base of well-being experiences in a way that is unique to the lived experiences of Africans (Adedeji et al., 2023). Such a gap in research is further aggravated by the use of instruments that were not designed for the African context (Oladele et al., 2024) as well as recurring weaknesses linked to validated instruments adopted from Western contexts.

African perspectives on well-being are grounded in relational, holistic, and communal ontologies that view individuals as embedded within social, spiritual, and ecological systems. As Wilson Fadiji and Khumalo (accepted) argue, African epistemologies emphasise interconnectedness, moral personhood, and contextual meaning-making dimensions that challenge Western individualistic models. These metatheoretical foundations shape how well-being is experienced and measured, underscoring the need for instruments that reflect African worldviews (Adedeji et al., 2023; Metz, 2017; Osei-Tutu et al., 2022).

Globally, well-being is understood as a dynamic, multidimensional construct situated within biocultural environments, encompassing positive feelings, functioning, quality of life, and resilience (Delle Fave et al., 2023). However, African conceptualisations extend beyond the hedonic-eudaimonic distinction to include constructs such as harmony, balance, and relational flourishing. Harmony is not merely an additional facet of well-being but reflects a broader worldview in which balance within oneself, one’s community, and the natural environment is central to flourishing (Delle Fave et al., 2023).

Interest in the measurement and promotion of well-being has increased exponentially, with calls for societal transformation and a new vision for health that places well-being at the centre (Plough, 2015). As research on well-being, both as an outcome (or a target for monitoring) and as a predictor of other health-related outcomes, has expanded dramatically, conceptions and measures of well-being have likewise proliferated. Consequently, it can be challenging to compare ideas and findings across different measures and conceptions. This is because the social-cultural context of those being assessed influences their well-being, and the use of culturally appropriate measures helps ensure the right levels of well-being are captured.

Conducting a systematic review would provide a comprehensive, unbiased synthesis of many relevant studies in a single document and may help uncover evidence relevant to understanding the extent of use, adaptation, and applicability of well-being instruments within the African context. In the current study, we build on the bibliometric and scoping review studies (Fadiji et al., 2024; Wilson Fadiji & Khumalo, 2026), demonstrating the current evidence on the advancement of well-being measurements in the African context while acknowledging the methodological gaps in the field with important implications for future research and practice of well-being.

### 1.1. Concept Clarification and Well-Being Measurement

At least three conceptual approaches to evaluating subjective well-being are commonly used: hedonic, evaluative, and eudaimonic conceptions (Kahneman & Krueger, 2006; Panel on Measuring SWB in a Policy-Relevant Framework et al., 2013; Ryff, 1989). A hedonic perspective focuses on well-being understood as whether one feels happy or experiences pleasure and lacks pain, as well as the degree to which an individual assesses the overall quality of her life-as-a-whole favourably. In contrast, an evaluative perspective focuses on well-being defined by one’s view of, or overall satisfaction with, life or different domains of life. A eudaimonic perspective focuses on whether individuals feel they have attained self-realisation or are fully functioning and fulfilling a sense of purpose. There is general agreement that well-being is a broad, multidimensional construct that extends beyond simply feeling happy or satisfied with life (Brandt et al., 2022; Stiglitz, 2019). Well-being can also be characterised by objective measures, also referred to as measures related to “standard of living”. Objective aspects of well-being will, of course, also influence subjective well-being levels (P. C. Patel & Wolfe, 2019).

In 2013, the Organisation for Economic Co-operation and Development (OECD) recognised the importance of considering people’s perceived well-being, or subjective well-being, when investigating overall well-being (Organisation for Economic Co-operation and Development, 2013). To assess subjective well-being, self-reports have typically been considered the most feasible approach. Self-report measures are reliable, providing accuracy and temporal stability that are valid for community surveys and cross-cultural comparisons, and can capture happiness as life-as-a-whole as well as domain satisfactions (Tay et al., 2014). Examples of international self-reported surveys are the Gallup World Poll (Deaton, 2008) and the World Values Survey (Easterlin, 2015; Easterlin & O’Connor, 2021), and the Gallup-Healthways Well-being index (Kahneman & Deaton, 2010). A definition of well-being not necessarily captured by these dimensions is an individual’s level of harmony or disharmony with the components of his/her cosmos or world. The presence of disharmony or disruption in the relationship between an individual and their cosmos leads to illness in the African context (Igbokwe & Ndom, 2008).

Most individual well-being measures are typically designed as self-report instruments and are usually developed in one context and then adapted for use in another. However, during further validation for use across new sociocultural contexts, some measures emerge as not ideal for fully capturing well-being experiences. A classic example is the transference of measures developed in the West and their further use in the African context. Despite the acknowledgement of the limitations of some of these measures across different empirical studies in this context (Ejoke et al., 2024), there has been no comprehensive review of these existing challenges or limitations or their implications for research in well-being, except for a literature review (Oladele et al., 2024).

### 1.2. Well-Being Measurement in Africa

The recent proliferation of studies on well-being has provided an exciting array of results and novel insights. However, there remain measurement and conceptual challenges, as there is little guidance on how existing measures adequately capture the unique socio-cultural experiences of Africans. In 2018, the African Journal of Psychological Assessment was released to publish scholarly work on assessments in Africa. Pertaining to well-being, as of February 2025, the 86 published articles since its inception included articles on the validation of the cognitive component of subjective well-being (Pretorius & Padmanabhanunni, 2022), the Dispositional Hope Scale (Khumalo & Guse, 2022), psychological well-being measures among South African adults (Mpondo et al., 2021), social well-being (Khumalo et al., 2021), engagement (Musenze & Mayende, 2020) and harmony in life (Cromhout et al., 2023). In other international journals, further reporting on the validation of well-being instruments in Africa has been published. Despite the proliferation of validation studies, researchers in Africa continue to raise concerns about the theoretical validity of these measures (Chukwuorji & Osondu, 2023).

For instance, among general well-being instruments, the mental health continuum has been validated over the years (Appiah et al., 2022; Schutte & Wissing, 2017). In both Schutte and Appiah’s studies, problems arose with the psychological well-being subscales, as items did not load properly onto them. Appiah et al. (2022) reported low reliability for the psychological well-being subscale and raised concerns about translation and cultural differences in the items measuring this subscale. Another criticism was the scale’s structure, which led their findings to point to a general psychological well-being factor. When it comes to language, Chukwuorji and Osondu (2023) indicated that although published translations of well-being instruments were increasingly widespread, the distribution of available studies was uneven, with very low published translation activity in East and North Africa and a preponderance of publications on translated instruments in South Africa. The authors suggest deriving population norms for relevant translations, increasing funding and other resources for translation projects, developing cross-national collaborations on translations, and making the translated versions of well-being instruments more accessible for other researchers.

One of the earliest attempts at instrument development was the Fortitude Questionnaire (Pretorius & Heyns, 1998) and the General Psychological Well-Being Scale (GPWS). In the case of the GPWS, however, the items were based on existing Western measures, and a handful of validation studies were conducted to investigate its usefulness for measuring well-being in Africa (Khumalo et al., 2010; Wissing & van Eeden, 2002). The Harmony-Disharmony Scale was also developed and validated in a Nigerian sample (Igbokwe et al., 2015). Quite recently, Symonds et al. (2022), in the Journal of Happiness Studies, shared findings on the development of a child and adolescent well-being scale in Sierra Leone from a social evolutionary perspective. This scale tapped into personal, teacher, family, and peer dimensions of well-being. According to Symonds et al. (2022), common questions include: Are you happy? Do you think people care about you? Do your teachers help you? Do you feel safe with your family? Do other children love you for who you are? The qualitative and quantitative results showed that children’s lived experiences of these indicators of well-being were shaped by the sociocultural structure of their communities.

Growth in the field of measurement is further reflected in validation studies of specific facets of well-being. For eudaimonic well-being, these studies include meaning in life (Schutte et al., 2016) in a sample of South Africans and among Ghanaian urban adults (Wilson Fadiji et al., 2021), social well-being (Khumalo et al., 2021), harmony in life (Cromhout et al., 2023), and hope (Khumalo & Guse, 2022). Smith (2018) also tested the Warwick-Edinburgh Scale for mental well-being and found support for its utility in a South African adult sample. The Questionnaire for Eudaimonic Well-Being (QEWB) was validated by Boshoff (2012); however, the assumption of unidimensionality within parcels was not satisfied, which suggested that these analyses could have masked multidimensionality (Schutte et al., 2013).

Validated hedonic well-being measures include the Positive and Negative Affect Schedule (PANAS) (Appiah et al., 2020a), Life Satisfaction in Ghana (Wilson Fadiji et al., 2021), and Togo (Sovet et al., 2016). Awosola and Sunday (2021) developed and tested a happiness scale; however, no further studies have been conducted to validate it in other samples (Awosola & Sunday, 2021). Despite the current appreciation for validating well-being instruments, there is a need to ensure they are suitable for measuring well-being outside the West. There is still a lack of a comprehensive overview of which instruments work and which do not in Africa. This study will explore which methodological choices have informed the validation studies and identify the teething problems affecting the use of the scales. This study would be the first of its kind and will provide the needed evidence for further theoretical grounding in the development of culturally and contextually relevant well-being tools.

### 1.3. The Present Study

The Gallup World Survey, Global Flourishing Study, World Happiness Report, and other international assessments of well-being have proven useful for providing comparative well-being data; however, they are based on original Western measures, some of which, as our review will show, do not adequately measure well-being in Africa. We recognise efforts made in Counted et al. (2024) and similar papers (Lomas et al., 2025) linked to the contextually relevant assessments of well-being. However, we believe the results of this review might demonstrate other measurement issues unique to the African context not already captured in these international surveys, making them less sensitive to the African context. To address the gap in obtaining a proper overview of how well-being is currently being measured in the African context, the present study aims to systematically review and collate evidence on the validation of well-being measures in Africa. The current study had the following objectives:

1. To explore the nature of well-being instruments validated for use in the African context; (the nature of well-being refers to the ways in which well-being is conceptualized, operationalized, and measured across diverse African populations, including the extent to which prevailing models capture culturally relevant dimensions).
2. To understand the contributions of well-being measurement studies in this context to the global discourse;
3. To investigate the different validation methodologies adopted in well-being studies in this context;
4. To investigate methodological challenges linked to the applicability of these scales for measuring well-being in the African context.

## 2. Materials and Methodology

We used the systematic review methods proposed by Littell et al. (2008) and the procedures for preparing a review outlined in the Preferred Reporting Items for Systematic Reviews and Meta-Analyses (PRISMA) guidelines (Moher et al., 2009; see Supplementary Materials). This method was chosen because the primary aim of the present review, was broader than evaluating the quality of individual measurement properties, and includes synthesising psychometric evidence, mapping the nature of well-being measurement in Africa, identifying methodological approaches used in validation studies, documenting measurement challenges, and examining the extent of instrument development within the continent. Some PRISMA items, such as effect measures, are not directly applicable to measurement-focused reviews.

Team members were involved in the planning and development of the review framework. The lead author and one graduate student met bi-weekly during the initial review process to establish inter-rater reliability. During the paper selection phase and writing of the review, all research team members communicated regularly through team meetings and email.

Terminologies that captured the concept of well-being included flourishing, life satisfaction, happiness, emotional and psychological well-being, and quality of life. The concept of validation was searched using the terms validation, psychometric properties, and instrument development. To locate tests and measures, we searched concepts including but not limited to tools and instruments. We also added Africa as a search term.

### 2.1. Inclusion and Exclusion Criteria

The focus of our review was to highlight instruments used to measure well-being in Africa. Inclusion and exclusion criteria were first developed at the level of the empirical study record (including the abstract and other information about the study) and then at the level of the instrument to determine if it measured positive psychological well-being. The research papers were selected according to the inclusion and exclusion criteria outlined below:

#### 2.1.1. Inclusion Criteria

1. Must be a quantitative study focused on the validation of a well-being instrument;
2. Must be conducted in Africa or with African samples;
3. Must have been published in English;
4. Any specific well-being facet to be included, e.g., meaning, harmony, etc.;
5. Must have the full text available.

#### 2.1.2. Exclusion Criteria

1. Quantitative studies without a clear validation aim;
2. Validation of physical wellness and psychological distress instruments; (these instruments were retained only when co-validated alongside a well-being measure in the same study).
3. Validation studies not including African samples.

### 2.2. Search Strategy

In consultation with librarians, the lead author developed a list of search terms and concepts to identify relevant literature on measures of mental well-being in Africa. To establish inter-rater reliability, two reviewers (the lead author and one graduate assistant) independently reviewed 50% of the final records and compared notes on inclusion and exclusion criteria. This process continued until 90% percentage agreement was achieved between reviewers. Although percentage agreement was used as a practical indicator of screening consistency, we recognise that it does not account for agreement occurring by chance. Future reviews should consider calculating Cohen’s kappa or related chance-corrected statistics to provide a more robust assessment of inter-rater reliability. Any identified disagreements were resolved through discussion. The databases searched included APA PsycInfo (via EBSCOhost), Medline, and Scopus.

**Figure 1:**
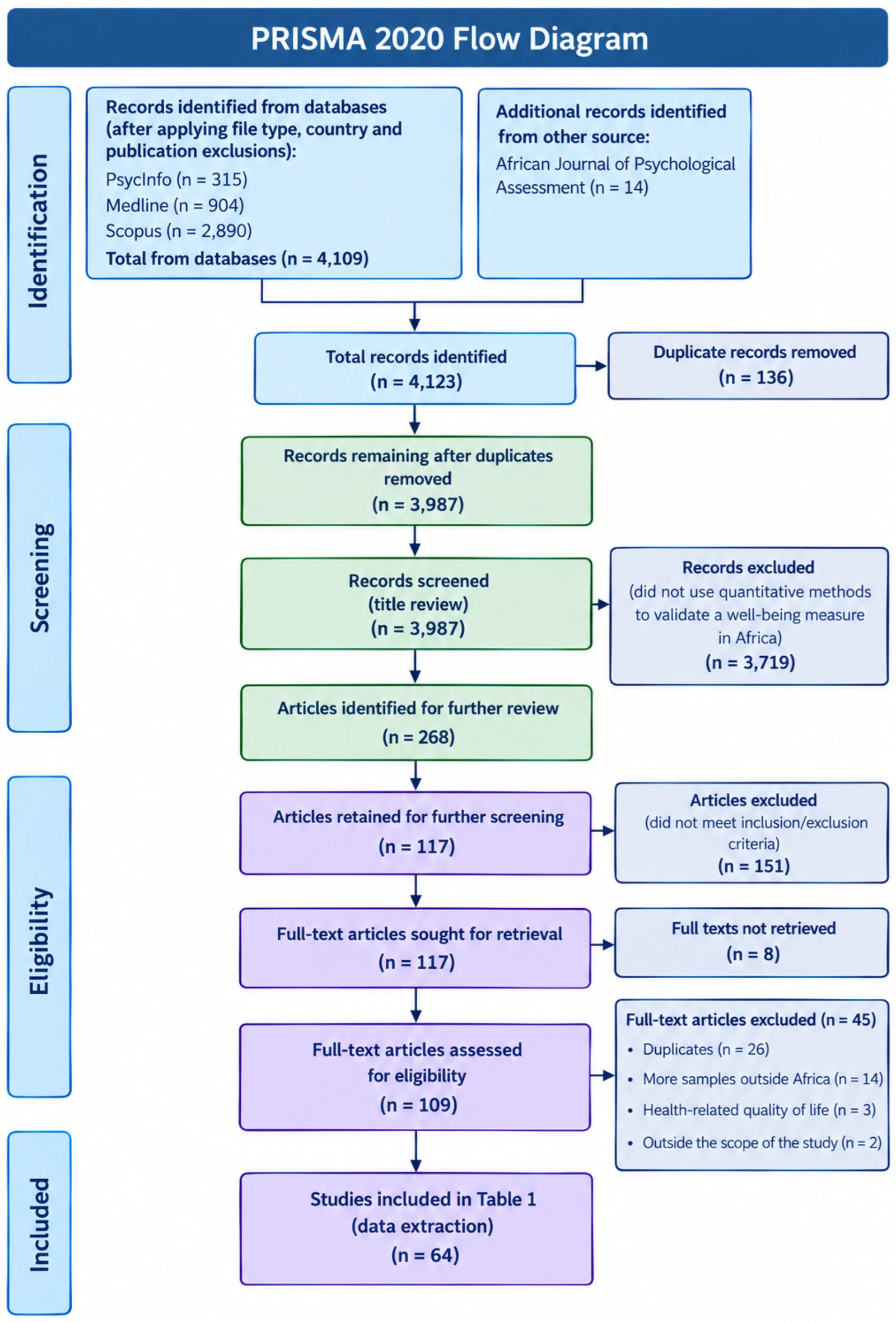
Flowchart of search strategy and systematic review process.

The search terms used in the review included: [Flourishing, OR life satisfaction, OR happiness, OR well-being, OR wellbeing, OR psychological well-being, OR quality of life, OR positive psychology] AND [instruments, OR validation, OR psychometric properties, OR instrument development, OR measurement] AND Africa. The search strategy used the following steps to select the final articles for the systematic review:

1. We used PsychInfo, Medline, and Scopus to conduct an initial search using the provided search terms. The initial results for quantitative articles in English were: PsycInfo (N = 315), Medline (N = 904), and Scopus (N = 2890). These results were obtained after excluding certain file types, countries, and publications (see Figure 1);
2. Given the focus of the African Journal of Psychological Assessment, we investigated the journal separately to determine whether there were other articles we might have missed. This search provided an additional 14 articles (N = 4123.)
3. In the next step, we exported the different outputs to Excel (N = 4123). After deleting duplicates (N = 136), the total number of articles was 3987. This list of articles was moved to the next stage of the analysis;
4. The research team met to review the initial output that emerged following the search that took place between April to May 2025. During this stage of the analysis, we applied the inclusion/exclusion criteria to screen titles and select articles to move to the next stage. After screening the titles, 3719 articles were excluded because they did not use quantitative methods to validate a well-being measure in Africa. The graduate assistant identified 268 articles that were moved to the next stage of the process.
5. Following this title, abstract, and full-text screening, studies were assessed against the other predefined inclusion and exclusion criteria. As illustrated in Figure 1, the screening process resulted in the retention of 117 studies (exclusion of 151 articles) for the final review. Figure 1 provides a complete account of records identified, screened, excluded, and retained at each stage of the review process.
6. The research team read through the full texts and completed Table 1 in response to the research questions. During the full-text reading, eight articles had to be excluded because we could not access their full texts. Several articles emerged as duplicates (26), and others did not meet the inclusion criteria because they included more samples outside Africa (14), focused on health-related quality of life (3), or were outside the scope of the study (2). A total of 64 articles were included in Table 1, capturing the development and validation of well-being instruments in Africa.

Figure 1 depicts a flowchart of the search strategy and systematic review process followed in the study.

A detailed overview of all 64 studies, including sample characteristics, instruments assessed, validation methodologies, methodological challenges, contributions, and study limitations, is provided in Supplementary Materials. Table 1 summarises the distribution of studies across major well-being domains.

## 3. Results and Discussion

The present study aimed to provide a systematic review of the evidence on the development and validation of well-being instruments within the African context. Specifically, we explored the nature of well-being instruments validated for use in this context, the key contributions to measuring well-being, the different validation methodologies adopted in well-being studies in this context, and the methodological challenges linked to the applicability of these scales in Africa. The results are presented according to the objectives and then discussed.

### 3.1. Objective 1: To Explore the Nature of Well-Being Instrument Development and Validation in Africa

A variety of psychological well-being, quality-of-life, and resilience scales emerged as having been tested in Africa. The evidence points mostly to validation rather than to the development of measurement scales, see Table 2 for a summary of the measures reviewed. Both eudaimonic measures and hedonic measures were highlighted as well as those capturing both dimensions of well-being including the Mental Health Continuum—Short Form (MHC-SF), Children’s World SWB and Psychological Well-being Scale, and the WHO Well-being Index. Resilience and Quality of Life Measures also form a separate dimension of well-being (See Table 2).

### 3.2. Objective 2: To Determine and Understand the Contributions of Validation Studies in the Region

Before highlighting the methodological challenges, we present examples of well-being measures that proved applicable for assessing well-being in African samples with minimal or no modification. For instance, the SWLS and the Mattering Scale (Stephen Lenz et al., 2018) were administered in the Ghanaian sample and found to be valid measures of these aspects of well-being, with αs of 0.72 and 0.64, respectively. In a different study, Appiah et al. (2020a) argued that the AFM, ATQ and GSE were the first validation studies of these measures conducted in Ghana and so provide useful information for subsequent studies (Appiah et al., 2020a). These measures were also translated into Twi, the language commonly spoken in Ghana. They emerged with omega-coefficients of 0.88, 0.98 and 0.97. Testing the SWLS in the same Ghanaian sample, the original unidimensional structure was upheld, demonstrating high internal consistency (0.87) and strong temporal stability over six months. Among South African children, the hierarchical model and four-factor scale of the children’s SWB showed a strong fit, with a reliability of 0.93, with expected correlations (Savahl et al., 2021).

In a different instance, apart from the need to remove Item 9 from the MLQ in a Ghanaian urban sample. Both MLQ and SWLS seemed suitable for measuring meaning (α = 0.84) and satisfaction with life (α = 0.84) (Wilson Fadiji et al., 2021). Furthermore, the 34-item Positive Emotion, Engagement, Relationships, Meaning, and Accomplishment (PERMA) Scale showed strong internal consistency across its five domains (above 0.70), aligning with the original measure and demonstrating reliability across cultures and in open- and distance-learning (ODL) settings with undergraduate students (Magare et al., 2022). In addition, the WHO-5 showed strong reliability (0.86) and validity, confirming its suitability for assessing mental well-being among healthcare professionals in Nigeria (Seb-Akahomen, 2022). The original three-dimensional structure of the MHC-SF (Keyes, 2002) provided the best model fit (α > 0.70), with full configural, convergent, and metric invariance across gender in a South African organisational context (Zyl & Olckers, 2019). Additionally, the psychological flourishing scale in an Arabic sample yielded high internal reliability and convergent validity evidence, provided initial temporal stability data and extended flourishing measurement into Egyptian/Arabic contexts (Salama-Younes, 2017).

Support was also found for the Flourishing at Work Scale. In a Moroccan and Algerian student sample, the Flourishing Scale demonstrated acceptable reliability. Additionally, measurement invariance analysis affirmed its robustness and validity for evaluating flourishing across varied cultural settings (Sabah et al., 2026). In the case of the Life Crafting Scale, it was suggested that Life Crafting is a deliberate process in which individuals create meaning by reframing their perspective, building social support, and pursuing growth through challenges (Chen et al., 2022). The original three-dimensional model of workplace spirituality, including meaningful work, community, and alignment with organisational values, emerged as key indicators of the construct (Kotze et al., 2022).

The first validation of the Afrikaans version of the Children’s Hope Scale demonstrated its reliability and validity for Afrikaans-speaking adolescents in South Africa. A one-factor model showed a good fit after incorporating two error covariances (Manuel et al., 2020). Linked to scale development, findings from the South African hope measure delivered an emic hope measure with five unidimensional scales, evidence of item invariance across gender/ethnic groups, and good item fit under Rasch analysis, illustrating how qualitative item development and Item Response Theory (IRT) yield culturally appropriate, psychometrically robust instruments (Maree et al., 2008).

The Swahili Connor-Davidson Resilience Scale-10 emerged as a reliable and valid measure of resilience, demonstrating strong internal consistency, construct validity, and stability across age and gender groups. It also effectively differentiated participants according to their resilience levels. Rasch analysis of the Connor–Davidson Resilience Scale further revealed a clear item hierarchy and confirmed the scale’s capacity to distinguish between varying levels of resilience. Consistent with this, both exploratory factor analysis (EFA) and confirmatory factor analysis (CFA) supported a unidimensional structure for the CD-RISC-10 (Mwangi et al., 2025). Similarly, the Resilience at Work Scale demonstrated strong internal consistency in a Global South context, reinforcing its cross-cultural psychometric robustness. The CFA supported a higher-order seven-factor structure, and measurement invariance testing, alongside multi-group path analysis, indicated no significant gender differences in workplace resilience. The scale also showed strong psychometric performance, integrating diverse theoretical dimensions of resilience—including interpersonal relationships, positive affect, psychosocial traits, cognitive strategies, mastery motivation, and stress inoculation (Sanhokwe & Takawira, 2022). Notably, it incorporated two novel components, early-life stability and toxic stress, to capture key psychobiological influences across the lifespan.

Measuring holistic well-being, the Quality-Of-Life Scale assessing housing and infrastructure, social relationships, SES, health, safety, and governance demonstrated good model fit and reliability, confirming its validity as a measure of quality of life within the Gauteng City region sample (Greyling & Tregenna, 2020). Other indicators of well-being, such as the Harmony in Life Scale, showed good fit except within a Setswana group (Cromhout et al., 2023). The ROT scores were reliable and exhibited construct validity in a large, religiously diverse South African student sample (C. J. Patel et al., 2013). Moving from the individual level, the abridged version of the Community Collective Efficacy Scale demonstrated acceptable reliability and construct validity in both urban and rural African samples and provided evidence that collective efficacy can be psychometrically measured in African contexts (van Straten et al., 2008).

Collectively, these findings suggest that the psychometric performance of wellbeing measures within African populations is variable and context dependent. While several instruments demonstrated acceptable reliability and evidence of validity, others showed poor model fit, inconsistent factor structures, or reduced reliability compared with findings reported in Western populations. These patterns highlight the importance of evaluating wellbeing measures within the cultural settings in which they are used, rather than assuming that psychometric properties will generalise across contexts. The findings also suggest that culturally relevant dimensions of wellbeing, such as communal relationships, social harmony, spirituality, and collective wellbeing, may not be fully captured by some existing instruments.

### 3.3. Discussion: Objectives 1 and 2

A common criticism of the field of positive psychology is the absence of evidence from contexts outside the West (Osei-Tutu et al., 2020; Wilson Fadiji et al., 2022). Although this might hold for some aspects of positive psychological research, the criticism might not accurately reflect well-being measurement. Our evidence points to a broad array of instruments being validated for use across the continent. These validations span multiple regions and demographic groups, demonstrating that tools reflecting the traditional hedonic-eudaimonic distinction as well as measures beyond this dichotomy (e.g., harmony) have been successfully applied and supported across diverse African samples. Collectively, these studies show that Africa-based validation work is more extensive than often assumed and that several instruments exhibit strong evidence of reliability, validity, and cultural applicability.

In support of the criticism, it is important to mention that only a few scales, such as GPW (Khumalo et al., 2010), SA Spirituality Scale (van Rensburg, 2020), and FORQ (Pretorius & Padmanabhanunni, 2020), were actually developed in the African context. What this implies is that we have more information on adaptability than on original measures fully informed by the African experience, world systems, and ways of knowing.

It is also necessary to point out the myriad studies on resilience and quality of life that emerge from our findings. In this context, the understanding of well-being seemed to encompass resilience and quality of life, probably because of adversities and positions of disadvantage that most Africans might find themselves. Including resilience and quality-of-life studies is one major difference between the present study and Fadiji et al. (2024), who excluded such studies in their bibliometric review. In the present instance, we include resilience and quality of life because international and cross-national measures of well-being tend to measure these indices as a way of assessing how happy or well a country is performing (Johnson & VanderWeele, 2022; Wilson Fadiji & Lomas, 2024). Therefore, understanding how these measures perform in this region of the world allows us to make some assertions about the reliability of the findings of existing international surveys.

Another noteworthy observation is the lack of further validation of locally developed well-being measuring instruments. Among them are the GPW and Spirituality Scale, and others developed in the South African context but not validated for use in other African countries or countries outside the continent. An exception is the FORQ that has been further validated in other studies (Pretorius & Padmanabhanunni, 2020; Yuwanto & Atmadji, 2017). This research gap likely accounts for the argument that there are no contextually relevant well-being instruments for use in Africa.

The evidence presented suggests a focus on eudaimonic indicators rather than affective dimensions of well-being. In Wilson Fadiji and Khumalo (2026), it was argued that qualitative research on the continent focused on eudaimonic indicators rather than emotional well-being. Such a focus captures the more traditional view of well-being where eudaimonic qualities of relational authenticity, spirituality and fulfilling normative expectations are prioritised (Joshanloo & Weijers, 2024). A focus on eudaimonic aspects reflects deep African cultural assumptions about the quality of relatedness among people, humans, and nature. The advent of modernity led to greater recognition, exploration, and expression of personal emotions, values, and insights as a guiding source for lifestyle choices and validation (Joshanloo & Weijers, 2024). It is possible that, despite modernity and urbanisation, which continually erode cultural values, a well-embedded value remains in placing importance on eudaimonic experiences. We are not arguing that hedonic measures do not perform well in the African context, but the exploration of eudaimonic indicators might reflect the dimensions of well-being core to the lived experiences of this sample. When linked to hedonic measures such as the SWLS, the reliability indices appear lower than those reported in studies outside the African context.

### 3.4. Objective 3: To Investigate the Different Validation Methodologies Adopted in Well-Being Studies

The third objective of the study was to determine which methodological approaches are being used, noting innovative analytical procedures and opportunities to advance knowledge of statistical techniques in well-being research. CFA was commonly used. This is expected given that most of these studies were designed to test the factor structure of existing scales. Exploratory factor analysis, including a test sample, has also been used. Other factor-analytic approaches that emerged included bifactor CFA, bifactor ESEM, ESEM models, and principal component analysis. Multigroup CFA and alignment optimisation are typically used to measure invariance across groups.

In some instances, the Classical Test Theory and IRT approaches were used as model-based latent variable techniques designed to examine the process by which individuals respond to items in a measurement instrument (Bean & Bowen, 2021). An assumption is made that each respondent has a position on the latent trait (such as social isolation) that influences the probability he or she will select a particular item response category (such as “No, never”). Other examples are latent class analysis and Rasch analysis. In a singular instance (Life Crafting Scale), a mixed-method approach was used. The development of the South African Hope measure was also based on findings from a qualitative study.

### 3.5. Objective 4: To Explore Methodological and Theoretical Challenges Linked to Current Measures of Well-Being

An objective of the present study is to critically examine the limitations of applying well-being measurement instruments developed in non-African contexts to African populations. In doing so, we identified several key methodological challenges.

#### 3.5.1. Translation Issues

Among the measures included in our review, a few were translated into local languages; however, some items on the scales did not seem to perform well after translation. An example is the BRS and the Grit Scale (Stephen Lenz et al., 2018). The authors argued that it is possible that, among the items removed from the BRS structural models, colloquial dialogue within assessment items may not have been readily transferred by their sample (Stephen Lenz et al., 2018). The Harmony in Life Scale, although demonstrating a good fit, was not replicated in the Setswana group, most likely due to translation issues, given that similar issues did not emerge in the English versions (Cromhout et al., 2023). It was argued that the exact meaning of some scale items was altered when the Harmony in Life (HILS) Scale was translated from English to Setswana. Specifically, there is no separate word for ‘harmony’ in Setswana. Harmony, which appears in Items 1 and 3 of the HILS, has been translated with the Setswana word ‘kagiso’, which means ‘peace’, and can refer to being neighbourly or getting along with others, rather than inner harmony, which may be the connotation with the English word in the context of the scale items’ phrasing (see Metz, 2017). The Mental Health Continuum in a sample of first-year students also showed only partial invariance among different language groups. It was argued that care needed to be taken when doing cross-language comparisons when using the MHC (Mostert et al., 2024).

#### 3.5.2. Poor Model Fit and Low Factor Loadings

The most recurring methodological challenge we observed in the validation of well-being instruments in Africa was poor model fit and low factor loadings. Given that CFAs are the most popular techniques for assessing whether the data from the well-being instrument fit the sample, it is not surprising that the biggest challenge lies in the fit indices and factor loadings. We provide specific examples of these in this section.

In a Ghanaian sample, the mental health continuum showed adequate fit only for the general mental health factor and poor loadings for psychological and social well-being factors (Appiah et al., 2022). The QEWB presented a similar challenge (Cromhout et al., 2022). In a very specific sample of tennis players, the Resilience Scale showed a rather poor model fit (Cowden et al., 2016). In most cases, the modification index was used to adjust the model to improve model fit.

The Human Spirituality Scale, designed to measure personal spirituality, showed a poor fit with the South African sample, necessitating the elimination of non-contributing items. It was argued that these challenges underscore the importance of revalidating and refining psychometric tools before applying them across different cultural contexts (van der Walt & de Klerk, 2014). The SWLS emerged with a poor-fitting model with problems in Items 4 and 5 following a Rasch analysis (Schutte et al., 2021). Other examples include a resilience at work scale, where only six of the seven latent factors met the threshold (Sanhokwe & Takawira, 2022), the SOC scale with an initial poor-fitting model (Getnet & Alem, 2019) and the Ryff’s Psychological Well-Being Scale showing a two-factor model where grouping positively and negatively worded items separately was the best fit for the South African sample, instead of the originally hypothesised model (Henn et al., 2016).

Validation studies of the MHC-SF conducted with African samples indicate that none of the tested measurement models demonstrated an adequate fit to the data. The findings indicate a qualitative distinction between the overarching well-being factor and the three specific subscales. Moreover, the low factor loadings and the corresponding omega coefficients suggest that the group-level factors lack sufficient reliability and precision for meaningful practical application (Opperman et al., 2025). A comparable issue has been observed in the validation of a resilience scale, where the seeking social support and enhanced personal resources factor consistently failed to demonstrate significant loadings. Furthermore, the combined correlated factors accounted for only 33% of the total variance, indicating limited explanatory power and raising concerns about the scale’s structural robustness (Strumpfer, 2001).

The FORQ also demonstrated variability in its psychometric behaviour across age groups. Among adolescents, responses reflected a dominant general factor, whereas adult samples supported a multidimensional structure. Notably, the subscales exhibited low reliability within the adolescent group, raising concerns about their developmental appropriateness (Pretorius & Padmanabhanunni, 2020). Similarly, validation work on the Social Well-Being Scale revealed substantial structural limitations: the intended five-factor model showed a poor fit, necessitating the removal of four items, and two of the remaining dimensions were classified as minor because each loaded on only two items (Khumalo et al., 2021). In a separate study conducted within a South African organisational context, the scale produced a three-factor structure, further underscoring the need for contextual re-operationalisation when applied in African workplace settings (de Jager et al., 2008).

Other measurement instruments have demonstrated similar validation challenges. For example, the Life Orientation Test (van Wijk, 2022) failed to support its theorised single-factor structure, indicating misalignment between the conceptual model and empirical data. Likewise, the three-factor structure of the Orientation to Happiness Questionnaire (Rothmann, 2013) did not replicate consistently across samples, with the engagement subscale emerging as particularly problematic. Across several studies, low or marginal reliability issues were reported, and in some cases, reliability information, such as test–retest estimates, was either limited or entirely absent. In the validation of the Wagnild and Young Resilience Scale, initial inter-item correlation analyses revealed that items 11, 20, and 22 did not correlate significantly with the overall scale and did not show meaningful associations with at least three other items. These weaknesses contributed to poor reliability and raised concerns about the measure’s structural integrity.

#### 3.5.3. Limited Sample

Beyond issues of poor model fit, a recurring methodological limitation across several studies is the use of small or narrowly defined samples, which restricts the generalisability of the findings. In some cases, researchers relied on non-random convenience samples or focused exclusively on specific age cohorts residing in urban areas. This limitation was evident in the validation of the MHC-SF, where results could not be generalised to less educated or older populations within the African context. Although the Children’s well-being—Subjective well-being and Psychological well-being scales employed relatively large samples, their validation found that the samples were not nationally representative, and language-related translation challenges compromised the validity of the instruments (Gentz et al., 2024). Similarly, the Child and Youth Resilience Scale relied on convenience sampling, which limits the replicability and generalisability of its findings (Govender et al., 2017). The Arabic adaptation of a Western flourishing scale was validated exclusively within a student population (Sabah et al., 2026), while the Spirituality Scale was tested using a homogenous sample of white Christian females, further restricting the applicability of the results (van der Walt & de Klerk, 2014). Although the PERMA Scale demonstrated strong internal reliability among distance learning students, the sample size was notably small (Magare et al., 2022). Additional examples include the Dispositional Hope Scale, which was validated solely with student samples, highlighting the need for cross-language and community-level replication (Khumalo & Guse, 2022). Likewise, the Satisfaction with life scale was tested only among teachers; despite the use of rigorous methodological procedures, its external validity across other occupational groups, age cohorts, and linguistic communities remains unverified (Pretorius & Padmanabhanunni, 2022).

### 3.6. Discussion of Objectives 3 and 4

Despite the proliferation of well-being instruments, VanderWeele et al. (2020) argue that the choice and suitability of a measure depend on the context. Translation and accurate cultural representation are key aspects of contextual consideration (VanderWeele et al., 2020). Translation will be useful to determine the extent to which certain conceptualisations of well-being are generalisable across cultures and groups (Chukwuorji & Osondu, 2023). The translation will reveal whether these constructs have indigenous dimensions not accounted for by Western-based theories or models (Cheung et al., 2011). In the present study, a number of measures, which were translated into African languages although deemed applicable for capturing the constructs they represent, had their shortcomings. For instance, the emergence of only partial invariance among language groups in South Africa (Mostert et al., 2024) points to the need to further explore the cultural meanings of well-being items. Psychological well-being items on the Mental Health Continuum (MHC) Scale in a rural sample of Ghanaian adults were argued not to translate properly into the Twi dialect and to require excessive introspection, which is not necessarily culturally congruent with Ghanaian values (Appiah et al., 2022).

Cooke et al. (2016) systematically searched databases for self-report instruments assessing well-being. They identified 42 instruments that varied significantly in length, psychometric properties, and their conceptualisations and operationalisations of well-being. They found a wide divergence across the different theoretical conceptualisations of well-being and how well-being is operationalised within particular theoretical categories. Only a few of these measures of well-being were developed and tested in multicultural samples, including non-Western cultures outside Europe, Australia or the United States (Cooke et al., 2016).

Diversity in the way well-being is conceptualised and measured is also reflected in the terms used to identify the various measures and their subscales. In some cases, different terms were used to refer to a very similar conceptualisation of well-being (e.g., the use of “happiness” appears indistinguishable from “life satisfaction” in the European Social Survey (2014). There appeared to be no distinction between the terms “quality of life” and “SWB” in the Quality of Life Inventory (Frisch et al., 1992). These have implications for the accurate measurement of different well-being constructs.

In their review of well-being instruments, Cooke et al. (2016) found that the reliability coefficients for several instruments were low and sometimes lower than what was recommended, even for research purposes. The evidence supporting the validity of several instruments was minimal, and many cases relied solely on face validity. In the present study, we found that several measures had low or marginal reliability. Others did not provide test–retest reliability, all of which are important for the use of these measures in a variety of settings.

A related and frequently observed methodological concern across the reviewed studies is the consistent emergence of poor model fit indices and weak factor loadings. Two primary validation approaches were commonly employed: CFA and Rasch analysis, and in several CFA applications, specific items failed to load onto their intended factors. It could be argued that the well-being instruments examined tend to emphasise intrapsychic dimensions of functioning, as noted by Cooke et al. (2016), while paying limited attention to contextual determinants of well-being, such as the ability to meet basic needs, financial adequacy, and the functioning of one’s family system. The omission of these domains may reflect a predominantly Western, individualistic conceptualisation of health and well-being. These conceptual and methodological limitations highlighted by Cooke may help explain the poor model fit indices observed in several of the reviewed measures. Notably, the Human Spirituality Index stands out as one of the few instruments that explicitly captures experiences likely to be salient within African contexts.

Dzokoto (2012) argues that understanding happiness in a place such as Ghana requires taking global, cultural, and phenomenological perspectives, moving from macro-structural factors to considerations of personal experience. Likewise, speaking specifically to Tanzania, Kilonzo and Simmons (1998) have argued that positive mental health in Tanzanian communities can only be understood in relation to traditional contexts where health involves a distinctive intertwining of spiritual, mental, social, and physical realms (Kilonzo & Simmons, 1998). These arguments suggest that measures of well-being that do not tap into this complexity in alternative worldviews might capture only a partial reality of individuals’ well-being in that context.

Prudon (2015) highlights several limitations of CFA, including its requirement for large sample sizes. This requirement is inherently complex, as a large sample may lead the χ^2^ statistic to indicate model misfit simply because of high statistical power, even when the model is substantively adequate (Prudon, 2015). At the same time, structural equation modelling (SEM) relies on large samples to function effectively, and goodness-of-fit indices similarly require substantial sample sizes to distinguish between well-fitting and poorly fitting models. However, such sample sizes are often unattainable in psychological research, particularly within clinical settings. There is also evidence that increasing the number of factors relative to the number of observed variables reduces the ability of indices such as the root mean square error of approximation and standardised root mean square residual to detect model misspecification.

Another concern relates not only to the technique itself but to how CFA is commonly applied. In some cases, researchers allow too many parameters to remain freely estimated, resulting in artificially inflated fit indices that offer limited interpretive value. Conversely, overly restrictive models—such as those that exclude theoretically justified cross-loadings or correlated error terms—may also compromise model accuracy. Prudon (2015) argues that more complete and theoretically precise measurement models are likely to yield more meaningful χ^2^ values and more reliable goodness-of-fit indices. In response to these challenges, the use of Exploratory Structural Equation Modelling (ESEM) has been recommended. Although some of the reviewed studies have adopted ESEM, many continue to rely exclusively on traditional CFA approaches.

## 4. General Discussion and Key Conclusions

The present study is a systematic review of the validation of well-being instruments in the African context. The objectives of the study include determining the nature and contribution of research in the African context, identifying different validation methodologies, as well as critically analysing the methodological limitations of current well-being instruments. Our findings point to steady growth in well-being measurement in the African context. However, this growth has not been without challenges that are stifling our general understanding and consensus on how well-being should be measured in different contexts, in this instance, Africa. This is because, generally, most validation research has been constructed in a top-down theory-driven way, rather than emerging from the ground up in an open, data-driven, inductive process (Lomas et al., 2026). We do acknowledge that these challenges are not necessarily unique to the African context, but they are insightful for what scholars need to be paying attention to when measuring well-being in a way that reflects the lived experiences of those being assessed.

Given the extensive coverage of well-being measures, our review showed that several hedonic and eudaimonic-related well-being indicators have been validated. We found more support, or at least more research, into eudaimonic-oriented and multidimensional well-being rather than purely hedonic measures. The SWLS, SLS, Affectometer and SWB were among the hedonic well-being measures identified. It is quite important to highlight the efforts made to validate multidimensional measures, as this clearly reflects the need to adequately capture well-being needs holistically across diverse contexts. In support of multidimensional well-being, data from across different parts of the world showed that balance and harmony, negative states, positive states, positive life appraisal, material security, societal safety, and character and virtue have emerged as likely universal factors or indicators of well-being (Lomas et al., 2026).

Moreover, the validation of resilience and quality-of-life measures as part of well-being in the African context is noteworthy. In Fadiji et al. (2024), resilience and quality of life were excluded from their bibliometric analysis of positive psychology because some of the underlying assumptions of resilience do not align with those of positive psychology. However, when it comes to measuring the lived experience of well-being in Africans who, on average, are faced by multiple adversities, the assessment of resilience and quality of life becomes paramount (Counted et al., 2024). Quality-of-life measures and the WHO’s well-being index are increasingly a core part of international surveys, given how closely a person’s well-being is tied to their social, economic, and spiritual environments. A notable string of literature is the Global Flourishing Study that continues to expand the index of well-being from life evaluation, daily emotions and quality of life to an expansive domain of well-being, including flourishing, psychological well-being, distress, physical health, financial well-being, and social well-being (Lomas et al., 2025). We found greater reliability for quality-of-life measures than for traditional positive psychology constructs.

Despite the steady growth we have alluded to, we are cognisant of the lack of progress in developing new, contextually relevant measures of well-being. The FORQ, GPW and Adolescent Well-being measures are among these few. We could speculate that the poor research in this regard is due to satisfaction with existing Western measures, the general lack of interest in such exploration, or the lack of funding support for this kind of work because its outcomes are seemingly limited to knowledge generation rather than impact or applied research. Irrespective of the reasons for the delay, we suggest that this is an important area of research in the African context. Data from international surveys are useful, but in-country analyses based on measures reflecting the context in question are useful for policy interventions and well-being practice.

### 4.1. Limitations

Systematic reviews are valuable for bringing together knowledge within a specific field; however, the credibility of the findings ultimately depends on the methodological rigour of the original studies. In the African context, the usefulness of existing measurement tools is therefore tied to the quality of the validation processes underpinning them. Although the procedures used in this review are transparent and replicable, the accuracy of the primary data cannot be independently verified within the scope of this study. A further limitation relates to access to research articles. Despite our efforts to be as comprehensive as possible by searching APA PsycInfo (via EBSCOhost), Medline, Scopus and AJOPA, some studies could not be included because their full texts were unavailable. We acknowledged the omission of Web of Science and AJOL as a limitation of the review and highlighted the need for future reviews to include these databases to maximise coverage of African scholarship. In addition, the English-only criterion itself likely contributes to the observed pattern of studies, as Francophone and Lusophone countries were poorly represented. Regarding the methods, although PRISMA guidance was followed, a formal COSMIN-based quality appraisal of included validation studies was not undertaken. Consequently, conclusions are based on psychometric findings as reported by the original studies and should be interpreted with appropriate caution.

### 4.2. Implications for Research and Practice

A clear implication of this study is the need for further testing of locally developed well-being instruments. These tools offer valuable insights, yet they also highlight gaps in the conceptual coverage of existing measures. This underscores the potential value of developing new, contextually grounded instruments. Although there is a substantial body of work on measurement, evidence of limited validity in some current tools suggests that there is still room for measures that better reflect local realities. Any new instruments must be accompanied by deliberate and ongoing investment in validation and re-validation processes. COSMIN guidelines can be adopted for future reviews that focus primarily on evaluating specific psychometric properties.

Furthermore, locally developed measures should inform or, at a minimum, be consulted during the construction of items for international surveys. Cross-country assessments can only generate meaningful insights when the items used adequately capture local experiences. We acknowledge that this is challenging, particularly given the need to balance comprehensive coverage with the practical constraints of survey length. Nonetheless, local researchers should be encouraged to use these indigenous instruments where appropriate. Doing so will enhance their visibility and generate additional data to strengthen evidence of their validity.

Expanding the translation of well-being measures is particularly important in countries with multiple officially spoken and written languages. Translation and measurement research deserve greater attention, as the knowledge generated has direct implications for well-being research and practice. Making well-being instruments available in local languages would facilitate their use in hard-to-reach communities and help capture cultural nuances that are often lost when English-only versions are used. This expansion would also support the adoption of such tools in clinical settings, especially as positive psychotherapy continues to gain traction.

Several scholars have argued for more intensive mixed-methods approaches as essential for deepening our understanding of local experiences of well-being (e.g., Thin, 2018; Strijker et al., 2020). These researchers emphasise that incorporating community perspectives is crucial not only as a complement to survey data, which can oversimplify the complexity of “culture,” but also as a humanistic commitment to honouring the diverse narratives that shape human experience.

Well-being interventions in both the community (Appiah et al., 2020b) and clinical settings (Walsh et al., 2017) are continuously gaining ground. However, the design and measurement of intervention outcomes cannot be properly grounded or adequately quantified if the measures being used do not capture the lived experience of African samples. Evidence of the impact of these interventions could further inform policy-related interventions targeted at improving well-being in the general population. More so, validated and contextually relevant measures can provide local indices on the levels of population well-being within a specific country and allow for the needed changes to improve well-being. Although not currently on the agenda of most African countries, measuring Gross National Happiness could be fostered when the right instruments for measuring well-being are available.

## Figures and Tables

**Table 1:** Summary of Well-Being Measurement Studies Included in the Review.

Construct Category	Example Instruments	Number of Studies	Development Studies	Validation Studies	Reported Reliability Range
Hedonic Well-Being	SWLS, Affectometer-2, Subjective Well-Being Scale, OHQ	10	0	10	0.72–0.93
Eudaimonic Well-Being	MLQ, PERMA, Flourishing Scale, GPWS, Hope, Spirituality, Social Well-Being, VIA character strength	28	5	23	0.64–0.98
Multidimensional Well-Being	MHC-SF, WHO-5, Warwick-Edinburgh Scale	11	0	11	0.70–0.90
Resilience	CD-RISC, Child and Youth Resilience Measure, Resilience at Work, Resilience Index	11	1	10	0.70–0.95
Quality of Life	Quality of Life Scale	1	1	0	Acceptable reliability reported
Collective and Community Well-Being	Community Collective Efficacy Scale	1	1	0	Acceptable reliability reported
Children’s Well-Being	Children’s SWB Scale, Children’s Psychological Well-Being Scale, Children’s Hope Scale	8	0	8	0.70–0.93
Total		64	8		

**Table 2:** Distribution of Well-Being Instruments Identified in the Review by Construct Category.

Construct Category	Representative Instruments	Number of Studies
Hedonic Well-Being	Satisfaction with Life Scale (SWLS), Affectometer-2, Subjective Well-Being Scale, Orientations to Happiness Questionnaire, Children’s Subjective Well-Being Scale	10
Eudaimonic Well-Being	Meaning in Life Questionnaire, PERMA Scale, Flourishing Scale, General Psychological Well-Being Scale, Life Regard Index, Hope Measures, Spirituality Measures, Social Well-Being Scale, Harmony in Life Scale, Workplace Spirituality Scale	28
Multidimensional Well-Being	Mental Health Continuum-Short Form, WHO-5 Well-Being Index, Warwick-Edinburgh Mental Well-Being Scale, Flourishing at Work Scale	11
Resilience and Quality of Life	CD-RISC, Resilience Scale, Child and Youth Resilience Measure, Resilience at Work Scale, Family Resilience Assessment Scale, Quality of Life Scale, Community Collective Efficacy Scale	15
Total	64 Studies	64

Note. Categories are not mutually exclusive. Some instruments assess multiple dimensions of well-being and were classified according to their primary conceptual focus.

## Data Availability

No new data were created or analyzed in this study. Data sharing is not applicable to this article.

## Supplementary Materials

The following supporting information can be downloaded at https://www.mdpi.com/article/10.3390/bs16101772/s1, PRISMA 2020 Checklist. Page et al. (2021) was cited in the Supplementary Materials.

## Abbreviations

AJOPA	African Journal of Psychological Assessment
BRS	Brief Resilience Scale
CFA	Confirmatory factor analysis
DHS	Dispositional Hope Scale
EFA	Exploratory factor analysis
ESEM	Exploratory Structural Equation Modelling
FORQ	Fortitude Questionnaire
GPW	General Psychological Well-Being
GPWS	General Psychological Well-Being Scale
GSE	Generalised Self-Efficacy
IRT	Item Response Theory
LRI	Life Regards Index
MHC	Mental Health Continuum
MLQ	Meaning in life Questionnaire
ODL	Open- and distance-learning
OECD	Organisation for Economic Co-operation and Development
OHQ	Orientations to Happiness Questionnaire
PANAS	Positive and Negative Affect Schedule
PERMA	Positive Emotion, Engagement, Relationships, Meaning, and Accomplishment
PRISMA	Preferred Reporting Items for Systematic Reviews and Meta-Analyses
PWB	Psychological Well-being
QEWB	Questionnaire for eudaimonic well-being
ROT	Religion Orientation Test
SASS	South African Spirituality Scale
SEM	Structural equation modelling
SOC	Sense of Coherence
SWB	Subjective Well-Being
SWLS	Satisfaction with Life Scale
